# Maximum Achievable N Content in Atom-by-Atom Growth of Amorphous Si-B-C-N Materials

**DOI:** 10.3390/ma14195744

**Published:** 2021-10-01

**Authors:** Jiri Houska

**Affiliations:** Department of Physics and NTIS—European Centre of Excellence, University of West Bohemia, Univerzitni 8, 30614 Plzen, Czech Republic; jhouska@kfy.zcu.cz

**Keywords:** N content, N_2_ formation, Si-B-C-N, Si-C-N, SiN_x_, BN_x_, CN_x_, C_3_N_4_

## Abstract

Amorphous Si-B-C-N alloys can combine exceptional oxidation resistance up to 1500 °C with high-temperature stability of superior functional properties. Because some of these characteristics require as high N content as possible, the maximum achievable N content in amorphous Si-B-C-N is examined by combining extensive ab initio molecular dynamics simulations with experimental data. The N content is limited by the formation of unbonded N_2_ molecules, which depends on the composition (most intensive in C rich materials, medium in B rich materials, least intensive in Si-rich materials) and on the density (increasing N_2_ formation with decreasing packing factor when the latter is below 0.28, at a higher slope of this increase at lower B content). The maximum content of N bonded in amorphous Si-B-C-N networks of lowest-energy densities is in the range from 34% to 57% (materials which can be grown without unbonded N_2_) or at most from 42% to 57% (at a cost of affecting materials characteristics by unbonded N_2_). The results are important for understanding the experimentally reported nitrogen contents, design of stable amorphous nitrides with optimized properties and pathways for their preparation, and identification of what is or is not possible to achieve in this field.

## 1. Introduction

Amorphous Si-B-C-N alloys are of high and long-term interest [1,2,3,4,5,6,7,8,9,10,11,12,13,14,15,16] because of unique combinations of functional properties such as high hardness [4,17], high optical transparency combined with controllable refractive index at some compositions [18,19] and controllable electrical conductivity at other compositions [20,21], strong absorption of electromagnetic waves [13,22], high photodetection sensitivity [23], strong photoluminescence [5], low leakage current [24] or giant piezorezistivity [14]. In parallel, proper Si-B-C-N compositions exhibit exceptionally high oxidation resistance up to 1500 °C [2,25], thermal stability of the amorphous networks up to 1700 °C [1,25], long-time (tested for 12 years) ageing resistance [19], low thermal expansion coefficient [2] and, perhaps most importantly, high-temperature stability of the aforementioned functional properties [18,25]. This makes the Si-B-C-N alloys very attractive for numerous applications ranging from high-temperature protective coatings to electronics and optoelectronics. The most recent examples include e.g., Si-B-C-N pressure sensors [14], Si-B-C-N protective coatings on optical fibers used at high temperatures [15] or Si-B-C-N-containing composites used for H_2_ production [16].

Bulk Si-B-C-N materials are often prepared by pyrolysis (recent review [10]) or mechanical alloying followed by sintering (recent review [12]). Thin films of Si-B-C-N can grow from isolated atoms (which is most relevant for the present work; usually using magnetron sputtering) [4,5,6,17,18,19], or from chemical precursors [15,26,27]. There have been numerous efforts to capture the role of individual elements and the relationships between their concentrations ([Si], [B], [C] and [N]_total—_not to be confused with [N]_network_ defined below; all given in at.%) and materials properties. See the works dealing with the [Si]/[C] ratio [19,28], [B] [28,29] or [N]_total_ [6,21]. For example, as much N as possible is desirable for high thermal stability, high hardness or wide band gap.

The status of nitrogen is special: while [Si], [B] and [C] can be varied from 0 to 100%, maximum achievable values of [N]_total_ are much lower and depend on the preparation technique (e.g., N_2_ pressure during sputtering [21] or combination of sputtering with N_(2)_^+^ ion beam [6]). See the literature overview [30] on CN*_x_* for an extreme case: maximum [N]_total_ obtained by various techniques of 23–45% (atom-by-atom growth) or 23–57% (including growth from N-rich precursors or assisted by N_2_^+^ bombardment). Furthermore, the composition is sometimes measured by techniques (e.g., X-ray fluorescence or elastic recoil detection) which cannot distinguish the content of N bonded in the amorphous networks ([N]_network_) and the total N content ([N]_total_ which may include also unbonded N_2_ or other N-rich molecules, reported e.g., for TiN [31] or oxidized TiN [32]). Thus, owing to the aforementioned importance of as high [N]_network_ as possible for some of the functional properties, it is desirable to quantify the highest achievable [N]_network_ at a given [Si]/[B]/[C] ratio.

The relationships between composition, density, structure and energy of (pure or hydrogenated) amorphous (Si)-(B)-(C)-(N) materials can be and are often studied by ab initio molecular dynamics (MD) simulations, see the examples for C [33,34], CH*_x_* [35], CN*_x_* [36,37], Si-C-H [38], Si-C-N [39,40] or Si-B-C-N [21,28]. The reliable predictions of local energy minima followed by their characterization in terms of bonding environment of individual atoms or localization of individual electronic states on these atoms allows one to obtain a lot of information not available experimentally. Recently, enhanced computing capacities have allowed the shift from modeling of few selected compositions to detailed sampling over a wide range of compositions and densities. 

The main aim of this work is to predict maximum achievable [N]_network_ in amorphous Si-B-C-N materials in a wide range of [Si]/[B]/[C] ratios. The background hypothesis, supported by the author’s previous works [30,41] on simpler nitrides and the discussion therein, is that maximum [N]_network_ in those materials which are not affected by the composition and structure of their precursors is largely given by the formation of N_2_ molecules during the atom-by-atom growth (not to be confused with polymerization of N-rich precursors such as melamine or dicyandiamide [42], which is beyond the present scope). Maximum [N]_network_ calculated at numerous [Si]/[B]/[C] ratios is compared with experimental [N]_total_ values obtained in our laboratory by reactive magnetron sputtering using equally numerous sputter target compositions. The effect of packing factor (not only atomic density) on the N_2_ formation is investigated as well.

## 2. Materials and Methods

### 2.1. Simulation Technique

All simulations utilized density functional theory (DFT) and Car–Parrinello MD implemented in the CPMD code [43]. The amorphous structures were predicted by the liquid-quench (LQ) algorithm consisting of (1) mixing of a melt (6000 K) of a given composition and density, (2) exponential cooling to a representative deposition temperature (450 K), (3) equilibration at this temperature and (4) collecting the results. The length of each step was 0.5 ps, leading to a total length of all steps of 2 ps. This frequently used [21,28,30,33,34,35,36,38,39,41] algorithm captures material formation conditions arising from melting and subsequent rapid quenching of a small volume of a material after an energetic particle impact. 

The presented energy differences (*E*-*E*_min_ where *E*_min_ corresponds to the lowest-energy density of a given composition) were obtained using time-averaged Kohn–Sham energies collected at the aforementioned temperature of 450 K. For the purpose of a low-temperature comparison of qualitatively similar structures of materials with a band gap, the relevance of time-averaged energy differences is considered to be comparable with that of free energy differences. The bonding statistics, including the number of N_2_ molecules ([N_2_]), were calculated using a representation of pairs of valence electrons by centers of maximally localized Wannier functions (WFCs) [38,44].

Further technical details of the simulations and detailed justification of the algorithm including its time scale, ranging from analytical calculation to the comparison of calculated bonding statistics with infrared spectroscopy, are included in the previous works [30,41].

### 2.2. List of Simulations

All simulations were performed with 70 atoms of random initial coordinates in a cubic periodical cell. The presented data span 16 different [Si]/[B]/[C] ratios including:

- [Si]/[Si+C] of 0, ≈11, ≈22, ≈33, ≈66 and 100% at [B] = 0,
- [B]/[B+C] of 0, ≈11, ≈22, ≈33, ≈66 and 100% at [Si] = 0 and
- [Si]/[Si+B+C] of, again, 0, ≈11, ≈22, ≈33, ≈66 and 100% at [B]/[C] ≈ 4.

The first group (B-free) is repeated for completeness from the previous work [41], the second and third group (B-containing) are considered for the first time. The [B]/[C] ratio of 4 which characterizes the third group is motivated by the experimentally used [18,19,21,25] B_4_C sputter target. 

**Figure 1:**
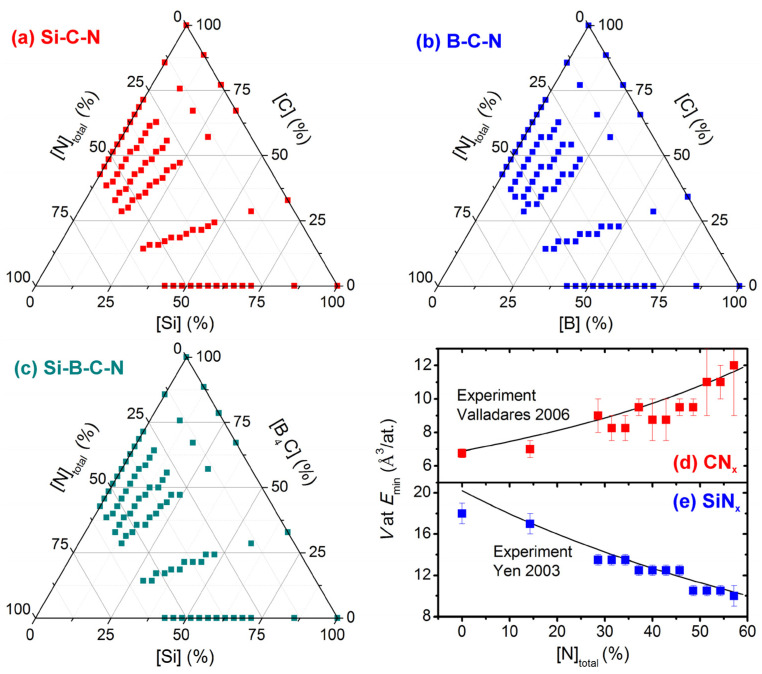
Panels (**a**–**c**) show the compositions simulated. There are 16 different [Si]/[B]/[C] ratios (18 lines of datapoints including SiN*_x_* and CN*_x_* which are shown in two panels) and 13 [N]_total_ values at each of these ratios. Some of the lines corresponding to B-containing compositions are more noisy because of keeping even number of electrons in the simulation cell. Panels (**d**,**e**) constitute a verification of the simulation technique and of the algorithm which allows the atoms to form unbonded N_2_. The datapoints show calculated lowest-energy inverse atomic density (*V*) of CN*_x_* (panel (**d**)) and SiN*_x_* (panel (**e**)) [41]. The black lines are fits to experimental densities of CN*_x_* (Equation (1) [37]) and SiN*_x_* (Equation (2) [45]). The error bars (mainly due to limited simulation cell size) represent the smallest intervals of *V* values which include simulations leading to (i) the two lowest energies and (ii) all other energies within 20 meV/at. from *E*_min_, if there are any.

Each [Si]/[B]/[C] ratio was combined with 13 [N]_total_ values up to 57%: 0, 10, 20, and then a step of 2 up to 40 N atoms in the 70-atom cell. All 16 × 13 = 208 compositions are visualized in Figure 1a–c. The total N content is given by (i) the number of unbonded nitrogen molecules ([N_2_]) and (ii) the content of nitrogen bonded in the amorphous networks, given in at.% out of the network atoms ([N]_network_).

Si-B-C-N materials with different energies and N_2_ contents were obtained using 13–19 inverse atomic densities (volumes per atom, *V*) of 6, 6.5, 7, 7.5, 8, and then a step of 1 up to 16, 18, 20 and 22 Å^3^/at. for [Si]/[Si+B+C] = 0–22%, 33–66%, 100% except pure Si and pure Si, respectively. These *V* ranges capture all lowest-energy densities calculated in this paper as well as all densities of crystalline or amorphous materials from the Si-B-C-N system found in the literature. Furthermore, the statistical noise has been reduced by performing each simulation 5× with different random initial coordinates and averaging all quantities (consequently, [N_2_] is not necessarily integer). Thus, the calculated data are based on 16 × 13 × 13 − 19 × 5 ≈ 15,000 liquid-quench simulations.

### 2.3. Reliability of the Simulation Protocol

While the LQ algorithm in itself is well established, it is worth discussing the decision to allow the N atoms to form unbonded N_2_ molecules which are not instantly lost to the atmosphere. Figure 1d,e show lowest-energy *V* of CN*_x_* and SiN*_x_* (binary systems chosen because of the availability of corresponding experimental data) predicted using the described simulation protocol in a wide [N]_total_ range. The calculated *V* is compared with measured *V* (converted from mass density, *ρ*, using the Avogadro number, N_A_, and the molar masses, M_Si,C,N_): 

*ρ*_CNx_ (g/cm^3^) = 2.897 − 0.01784 × [N]_total_ (fit on experimental data by authors of [37]) ⇔

*V*_CNx_ (Å^3^/at.) = {M_C_×(100 − [N]_total_) + M_N_×[N]_total_)}/{(2.897 − 0.01784 × [N]_total_) × N_A_ × 10^−22^} (1)

and

*ρ*_SiNx_ (g/cm^3^) = 2.3 + 0.01575 × [N]_total_ (own fit on experimental data reported in [45]) ⇔

*V*_SiNx_ (Å^3^/at.) = {M_Si_ × (100 − [N]_total_) + M_N_ × [N]_total_)}/{(2.3 + 0.01575 × [N]_total_) × N_A_ × 10^−22^}. (2)

It can be seen that the agreement shown in Figure 1d,e is excellent, supporting the correctness of the simulation protocol in general and the decision concerning allowed presence of unbonded N_2_ in particular. Indeed, *V* of CN*_x_* increases with [N]_total_ because of more/larger voids occupied by unbonded N_2_ (not e.g., because of covalent radii of both elements: that of N is actually slightly lower). It is crucial that the agreement was achieved without any fitting parameters, only by reproducing the material formation process on the experimentally relevant time scale. 

### 2.4. Reactive Magnetron Sputtering

The presented Si-B-C-N films were deposited on Si substrates by dc reactive magnetron sputtering of the following 15 composite targets [4,19,46]:

- Si*_x_*C_100-*x*_ (graphite overlapped by Si plates) at *x* = 5, 20, 40, 60 and 80%;
- Si*_x_*B_20_C_80-*x*_ (graphite overlapped by Si and B plates) at *x* = 5, 40, 60 and 75%; and
- Si*_x_*(B_4_C)_100-*x*_ (B_4_C overlapped by Si plates) at *x* = 0, 5, 20, 40, 60 and 75%.

The total pressure was 0.500 Pa and the composition of the discharge gas mixture (except the series which examines its effect) was 50% (0.250 Pa) N_2_ + 50% (0.250 Pa) Ar. Varying the substrate surface temperature (up to 700 °C) and substrate bias voltage (down to −500 V) did not visibly affect the measured [N]_total_ values (the effect of both these parameters on other film characteristics such as content of implanted argon, stress or hardness is examined elsewhere [17,19]). 

The film composition was measured by Rutherford back-scattering spectroscopy (evaluated by the code GISA [47]; all elements except H) and elastic recoil detection (evaluated by the code SIMNRA [48]; H) using a Van de Graaf generator with a linear electrostatic accelerator. The accuracy of compositional measurements was 1–2%. The constitutive elements were accompanied by small contents of Ar (≤5 at.%), H (≤5 at.%) and O (≈1 at.%), at a median [Ar+H+O] of 7%. These impurities were neglected for the present purpose, i.e., the presented [N]_total_ values are equal to [N]/[Si+B+C+N] rather then to [N]/[Si+B+C+N+Ar+H+O]. The effect of implanted Ar is investigated elsewhere [49].

## 3. Results and Discussion

### 3.1. Maximum Achievable N Content

**Figure 2:**
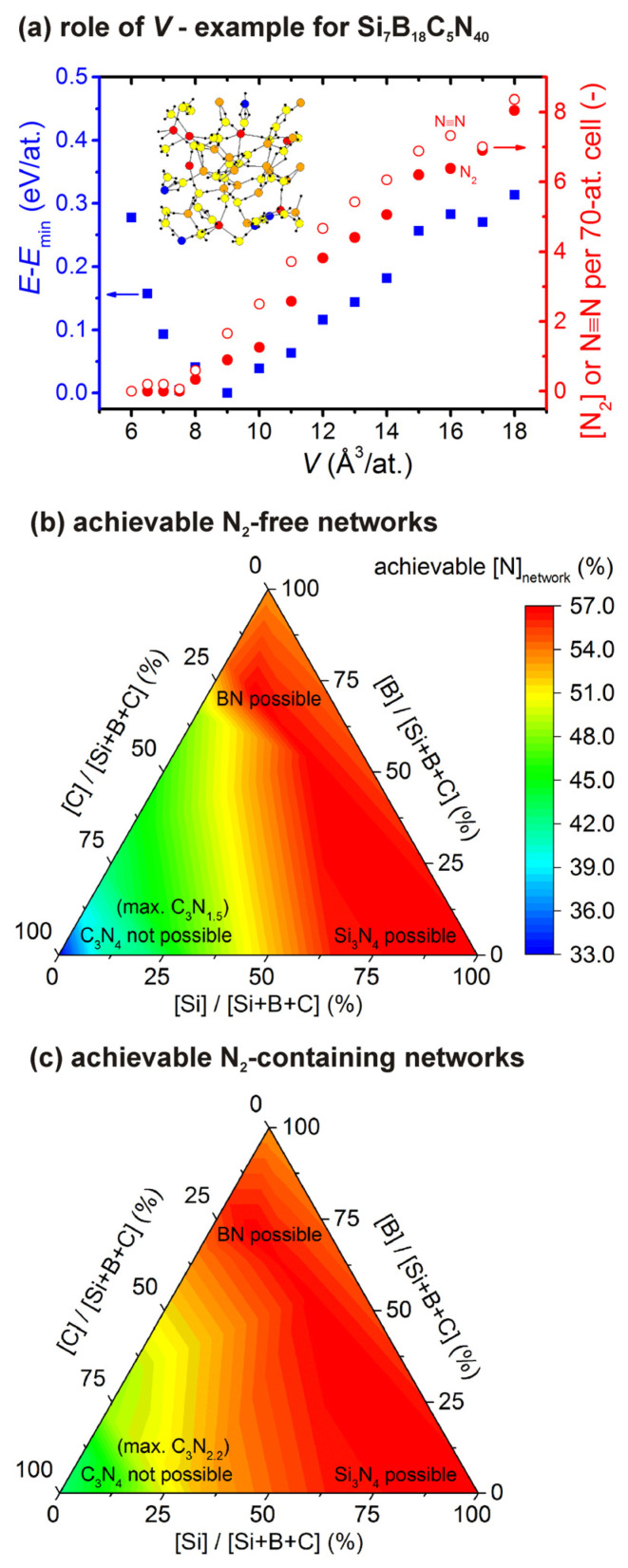
Panel (**a**) shows an example of the dependence of energy (*E*-*E*_min_; squares; left axis), number of unbonded N_2_ molecules ([N_2_]; full balls; right axis) and number of triple N≡N bonds (empty balls; right axis) on the inverse atomic density (*V*) for the composition of the 70-atom cell Si_7_B_18_C_5_N_40_. The inset is a snapshot taken at *V* = 9 Å^3^ (red Si, orange B, blue C, yellow N, small black WFCs). Panels (**b**,**c**) show the maximum achievable content of N bonded in Si-B-C-N networks ([N]_network_) without unbonded N_2,_ i.e., at [N]_network_ ≈ [N]_total_ (less than one N_2_ per cell; panel (**b**)) and at a presence of unbonded N_2,_ i.e., at [N]_network_ < [N]_total_ (panel (**c**)).

The N_2_ formation in itself and its dependence on *V* is illustrated in Figure 2a. The figure constitutes an example for the composition Si_7_B_18_C_5_N_40_ characterized by medium [Si]/[B]/[C] (complementary to extremal compositions Si_30_N_40_ and C_30_N_40_ discussed in detail previously [41]) and high [N]_total_. The figure shows a non-monotonic evolution of *E* characterized by *E* = *E*_min_ around *V* = 9 A^3^/at. This is combined with monotonically increasing [N_2_] from 0 at the highest densities (lowest *V*) to ≈8 at the lowest density (highest *V*) considered. Note that the number of triple N≡N bonds is only slightly higher than [N_2_], i.e., that most of N≡N bonds which are natural precursors of N_2_ formation indeed converted to unbonded N_2_ molecules. The lowest-energy density corresponds to a low but convincingly non-zero [N_2_]: the first fingerprint of the differences between [N]_total_ and stable [N]_network_ (Section 2.2) which are discussed next. 

Calculating lowest-energy [N_2_] and [N]_network_ for all elemental compositions investigated reveals (not shown graphically) that at fixed [Si]/[B]/[C], [N_2_] is almost zero up to a certain [N]_total_ threshold. Above this threshold, [N_2_] starts to linearly increase with [N]_total_ (most of the extra N atoms end up in unbonded N_2_ molecules) and [N]_network_ almost saturates. This allows one to predict the maximum achievable [N]_network_ as a function of [Si]/[B]/[C], and the result is shown in Figure 2b,c. Figure 2b shows the maximum [N]_network_ which is achievable without the presence of N_2_ molecules (less than one molecule per the 70-atom simulation cell). It can be seen that the predicted maximum [N]_network_ smoothly changes with [Si]/[B]/[C], including ≈34% for CN*_x_*, just above 50% for BN*_x_* (allowing BN) and 57% for SiN*_x_* (allowing Si_3_N_4_). The first of these numbers corresponds to C_3_N_1.5_, far from C_3_N_4_ which was predicted to be superhard in its *β* phase [50] but which seems to be impossible to prepare at least by the low-pressure atom-by-atom growth. The N-rich compositions allowed by the figure include SiBCN_3_ in the middle of the triangle, one of the early examples [2] of extremal thermal stability and oxidation resistance of Si-B-C-N ceramics. Figure 2c shows the aforementioned saturation values of [N]_network_ which are achievable when the network formation is accompanied by N_2_ formation. These values are of course higher than those in Figure 2b, e.g., ≈42% (C_3_N_2.2_) instead of ≈34% (C_3_N_1.5_) in the case of CN*_x_*, but arguably at a cost of properties affected by the lower densification resulting from the presence of voids occupied (either permanently, or temporarily until a loss to the atmosphere) by unbonded N_2_.

The dependence of the maximum achievable [N]_network_ on ratios of the other elements can be captured well by the following two fitted (*R*^2^ = 0.96 in both cases) quadratic dependencies on *x* = [Si]/[Si+B+C] and *y* = [B]/[Si+B+C]:

[N]_network_ (%) = 34.3 + 50.8*x* + 36.9*y −* 28.5*x*^2^ *−*16.9*y*^2^ *−* 33.9*xy* (3)

for networks without N_2_ (Figure 2b) and

[N]_network_ (%) = 41.7 + 39.1*x* + 31.5*y −* 24.3*x*^2^ *−* 18.7*y*^2^ *−* 37.1*xy* (4)

for networks with N_2_-containing voids (Figure 2c). 

This constitutes the key result of the present paper, which can be tested experimentally (below).

### 3.2. Densification and Packing Factor

**Figure 3:**
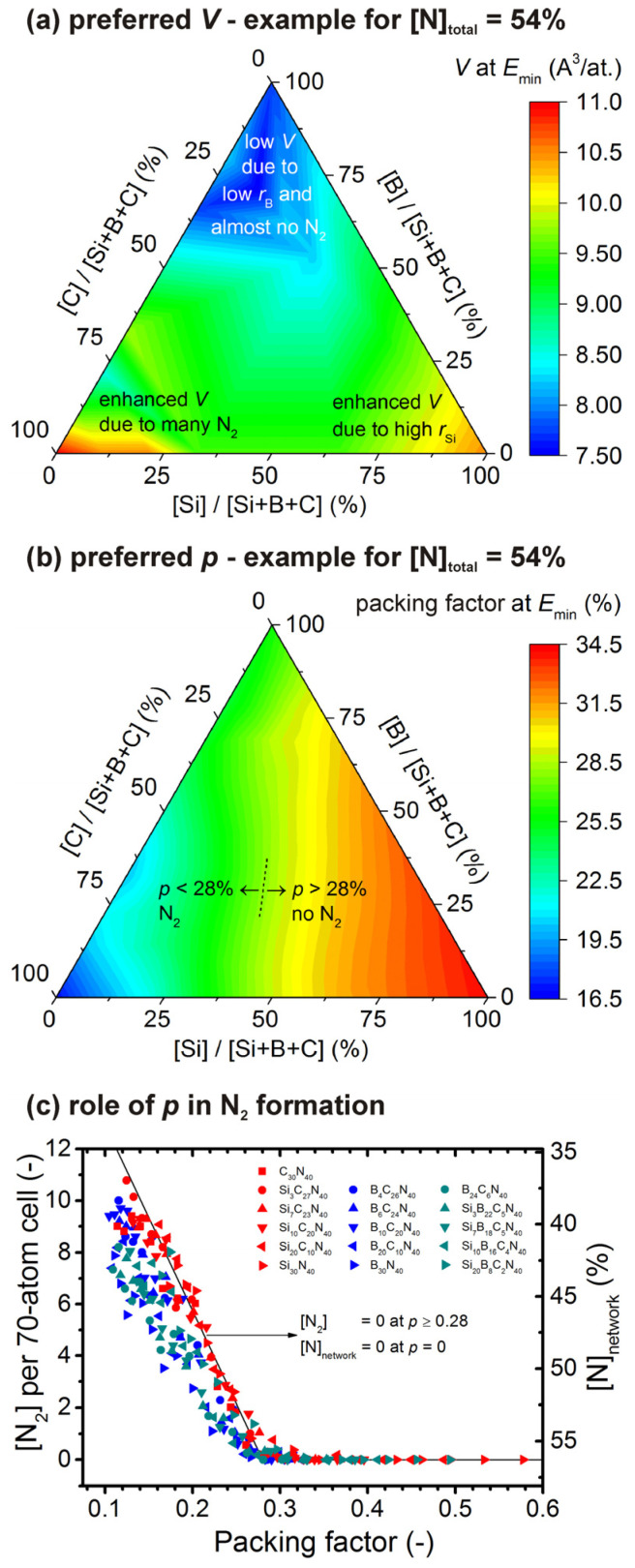
Panels (**a**,**b**) show lowest-energy inverse atomic density (*V*) and packing factor (*p*), respectively, of Si-B-C-N materials. The panels constitute an example for [N]_total_ = 54%, leading to similar *V* of CN*_x_* (which increases with [N]_total_—Figure 1d) and *V* of SiN*_x_* (which decreases with [N]_total_—Figure 1e). Panel (**c**) shows the N_2_ formation in terms of the number of unbonded N_2_ molecules ([N_2_], left axis) and the resulting content of N which remains bonded in the Si-B-C-N network ([N]_network_, right axis) as a function of *p*. The datapoints are shown for all 16 [Si]/[B]/[C] ratios, the highest [N]_total_ value and all *p* (⇔ *V*) values considered. The black line connects [N]_network_ = 0 (maximum [N_2_]) at *p* = 0 and [N_2_] = 0 (maximum [N]_network_) at *p* = 0.28 (critical value almost independent of [Si]/[B]/[C] and [N]_total_).

The formation of N_2_-containing voids affects the lowest-energy atomic density and packing factor (*p*; calculated using the covalent radii *r*_Si_ = 1.11 Å, *r*_B_ = 0.82 Å, *r*_C_ = 0.77 Å, *r*_N_ = 0.75 Å) of the materials. An example of the dependence of these two quantities on [Si]/[B]/[C] at a fixed [N]_total_ (shown for [N]_total_ = 54%, chosen because it leads to similar *V* of CN*_x_* and SiN*_x_*) is presented in Figure 3a,b, respectively. Indeed, Figure 3a shows that *V* of 10.5–11.0 Å^3^/at. can be found in the two bottom corners, i.e., for CN*_x_* and SiN*_x_*. However, as confirmed in Figure 3b, the reasons behind these two maxima are fundamentally different. In the case of CN*_x_*, the enhanced *V* (despite the low radii of C and N) is due to the large volume occupied by N_2_-containing voids and leading to a low packing factor down to ≈16%. In the case of SiN*_x_*, there are no unbonded N_2_ molecules which allows more than 2× higher packing factor of ≈34%, and the enhanced *V* is due to the high radius of Si atoms. Contrary to CN*_x_* and SiN*_x_* which represent local maxima of *V*, BN*_x_* represents the minimum *V* of ≈7.5 Å^3^/at. resulting from a combination of medium packing factor (nonzero but low number of N_2_ molecules) of ≈25% with the low radius of B. 

While the minimum *V* leading to N_2_ formation is different for different compositions, the maximum *p* leading to N_2_ formation is almost independent of the composition. In particular, Figure 3c shows that *p* ≥ 0.28 leads to almost zero [N_2_], while *p* < 0.28 leads to increasing [N_2_] with decreasing *p*. Note that while this finding is shown graphically only for the highest [N]_total_ investigated, it is independent of [N]_total_. The limit *p* ≥ 0.28 is consistent with the densities of covalent nitrides such as β-Si_3_N_4_ (*p* = 0.33), β-C_3_N_4_ (predicted *p* = 0.30) or c-BN (*p* = 0.34; the bonds between hexagonal planes of h-BN with *p* = 0.21 are not covalent). The results obtained at [B] = 0 almost exactly reproduce the linear dependence which connects [N_2_] = 0 at *p* = 0.28 with the other obvious limit [N]_network_ = 0 (nitrogen only in the form of N_2_ molecules) at *p* = 0. The results obtained at [B] > 0 exhibit the same critical *p* = 0.28, but lead to slower increase of [N_2_] with *p* decreasing below 28%, by a factor which is actually very close to (1 − 0.01 × [B]). This indicates, beyond the present scope, a qualitative difference between compositionally stable low-density B doped by N ([B] close to 100%, i.e., 1 − 0.01 × [B] close to zero) and unstable low-density Si or C doped by N. 

### 3.3. Experimental Verification

**Figure 4:**
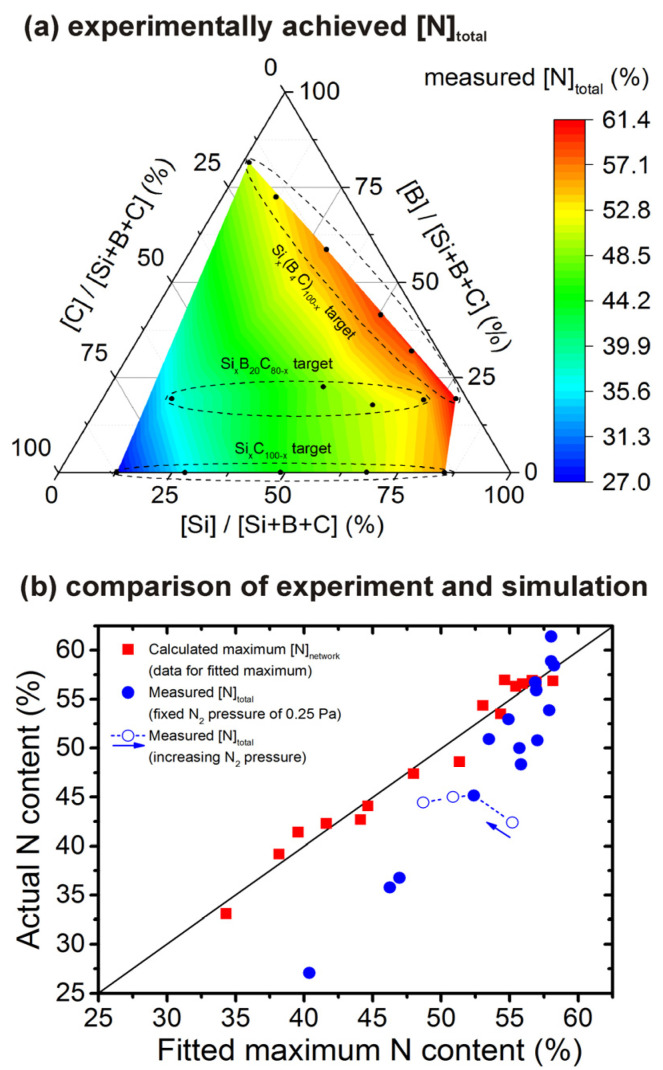
Panel (**a**) shows experimental values of [N]_total_ in Si-B-C-N (neglecting the impurities) prepared by reactive magnetron sputtering in 0.250 Pa of N_2_ + 0.250 Pa of Ar. The black balls correspond to compositions obtained using Si*_x_*C_100-*x*_ targets [46], Si*_x_*B_20_C_80-*x*_ targets [4] and Si*_x_*(B_4_C)_100-*x*_ targets [19] (bottom, middle and top dashed oval, respectively). Panel (**b**) compares these experimental values with those given by Equation (3). The squares are calculated maxima of [N]_network_ (Figure 2b). The diagonal line represents Equation (3) (fitted using these calculated data). The balls are the aforementioned values of [N]_total_ in Si-B-C-N (neglecting the impurities) obtained by reactive magnetron sputtering in 0.250 Pa of N_2_ + 0.250 Pa of Ar using Si*_x_*C_100-*x*_, Si*_x_*B_20_C_80-*x*_ and Si*_x_*(B_4_C)_100-*x*_ targets (full balls [4,19,46]) or in 0.125–0.500 Pa of N_2_ at a fixed total pressure of 0.500 Pa using the Si_40_C_60_ target (empty balls [53]; this dependence is not vertical because of different ratios of sputtering yields of individual elements by N_2_^+^ and by Ar^+^).

The experimentally measured [N]_total_ obtained by sputtering 15 different targets at fixed process parameters such as total N_2_+Ar pressure, N_2_ partial pressure or sputtering current, are shown in Figure 4a. Note that the experimental [N]_total_ values were calculated for this purpose as [N]/[Si+B+C+N] (27–61%) after neglecting the impurities, i.e., they are higher than the truly measured [N]/[Si+B+C+N+Ar+H+O] (26–54%). The figure conservatively spans only the pentagon defined by the compositions investigated, without any extrapolation. While the chosen deposition conditions do not necessarily always lead to the achievable maximum of [N]_total_ (see the quantitative comparison below), it is clear that the experimental data exhibit exactly the same trend as the theoretical prediction: lowest values of predicted maximum [N]_network_ and measured [N]_total_ in the C-rich corner, medium values in the middle of the triangle and in the B-rich corner, highest values in the Si-rich corner. In other words, the figure supports the predicted dependence of the N_2_ formation at the lowest-energy density on the composition. 

The qualitative agreement of the simulation (Figure 2b) and the experiment (Figure 4a) is complemented by their quantified comparison in Figure 4b. First, Figure 4b confirms that the calculated values of maximum [N]_network_ shown in Figure 2b are well represented by the simple quadratic Equation (3): the median vertical distance of squares in Figure 4b from the diagonal line is less than 1% of N. Second, the figure confirms that the measured [N]_total_ almost monotonically increases with the predicted maximum [N]_network_. Third, quantitatively, the measured [N]_total_ values are well below the predicted maxima when the prediction is low, but converge to the predicted maxima when the prediction is high. In other words, the figure shows that less intensive N_2_ formation and loss does not only mean that the maximum N content is higher, it also means that this higher N content can be achieved in a wider range of experimental conditions. The single [N]_total_ value which exceeds the predicted maximum [N]_network_ may not only be due to the presence of unbonded molecules (unlikely in this particular case, taking the thermal stability [25,28] into account) but also due to a higher than estimated measurement error (the four rightmost datapoints include two almost exactly reproducing the predicted maximum, one above it and one below it) and/or the role of impurities (e.g., compressive stress and changes in network topology resulting from implantation of Ar^+^ ions [49]: even when [N]/([Si+B+C+N] = 61% is above the prediction, [N]/[Si+B+C+N+Ar+O+H] = 54% is below it). The role of impurities is important in various field of materials science (e.g., [51,52]) but beyond the present scope. Fourth, the empty balls in Figure 4b confirm that increasing the N_2_ partial pressure from 0.125 Pa to 0.500 Pa can move the measured [N]_total_ from a value well below the prediction almost all the way toward the prediction.

## 4. Conclusions

Ab initio simulations of structures and energies of amorphous Si-B-C-N materials have been performed in a wide range of compositions and densities, and compared with the preparation of the same materials in the form of thin films by reactive magnetron sputtering in a wide range of sputter target compositions. The maximum content of N bonded in the Si-B-C-N networks is limited by the formation of unbonded N_2_ molecules, and can be well represented by a quadratic dependence (for N_2_-free materials; in %) 34.3 + 50.8*x* + 36.9*y* − 28.5*x*^2^ − 16.9*y*^2^ − 33.9*xy* where *x* = [Si]/[Si+B+C] and *y* = [B]/[Si+B+C]. While various compositions correspond to various lowest-energy packing factors both below and above 0.28, the N_2_ formation is non-negligible when the packing factor is below 0.28 regardless of the composition. The driving force toward N_2_ formation at a given packing factor (lower than 0.28) decreases with increasing B content. The theoretical densities and especially the maximum N contents (predicted without any fitting parameters, only by reproducing the material formation process) are in very good agreement with the experiment. Because as high N content as possible is crucial for numerous functional properties of Si-B-C-N, the results are useful for the design of these technologically important nitrides (including knowing when the content of bonded N can be enhanced without any tradeoff/can be enhanced at a cost of unbonded N_2_/cannot be enhanced), their preparation (e.g., conditions for N_2_ implantation and diffusion), knowing when to use characterization techniques (e.g., vibrational spectroscopy) which can distinguish bonded and unbonded N, and identification of what is or is not possible to achieve (including preparation of crystalline phases such as β-C_3_N_4_) in this field.

## Data Availability

The data that support the findings of this study are available from the author upon reasonable request.
